# Dexrazoxane Diminishes Doxorubicin-Induced Acute Ovarian Damage and Preserves Ovarian Function and Fecundity in Mice

**DOI:** 10.1371/journal.pone.0142588

**Published:** 2015-11-06

**Authors:** Jenna Kropp, Elon C. Roti Roti, Ashley Ringelstetter, Hasan Khatib, David H. Abbott, Sana M. Salih

**Affiliations:** 1 Department of Animal Sciences, University of Wisconsin, Madison, Wisconsin, United States of America; 2 Department of Obstetrics and Gynecology, University of Wisconsin, Madison, Wisconsin, United States of America; 3 Department of Medicine, University of Wisconsin, Madison, Wisconsin, United States of America; 4 Wisconsin National Primate Research Center, Madison, Wisconsin 53715, United States of America; University of Quebec at Trois-Rivieres, CANADA

## Abstract

Advances in cancer treatment utilizing multiple chemotherapies have dramatically increased cancer survivorship. Female cancer survivors treated with doxorubicin (DXR) chemotherapy often suffer from an acute impairment of ovarian function, which can persist as long-term, permanent ovarian insufficiency. Dexrazoxane (Dexra) pretreatment reduces DXR-induced insult in the heart, and protects *in vitro* cultured murine and non-human primate ovaries, demonstrating a drug-based shield to prevent DXR insult. The present study tested the ability of Dexra pretreatment to mitigate acute DXR chemotherapy ovarian toxicity in mice through the first 24 hours post-treatment, and improve subsequent long-term fertility throughout the reproductive lifespan. Adolescent CD-1 mice were treated with Dexra 1 hour prior to DXR treatment in a 1:1 mg or 10:1 mg Dexra:DXR ratio. During the acute injury period (2–24 hours post-injection), Dexra pretreatment at a 1:1 mg ratio decreased the extent of double strand DNA breaks, diminished γH2FAX activation, and reduced subsequent follicular cellular demise caused by DXR. In fertility and fecundity studies, dams pretreated with either Dexra:DXR dose ratio exhibited litter sizes larger than DXR-treated dams, and mice treated with a 1:1 mg Dexra:DXR ratio delivered pups with birth weights greater than DXR-treated females. While DXR significantly increased the “infertility index” (quantifying the percentage of dams failing to achieve pregnancy) through 6 gestations following treatment, Dexra pretreatment significantly reduced the infertility index following DXR treatment, improving fecundity. Low dose Dexra not only protected the ovaries, but also bestowed a considerable survival advantage following exposure to DXR chemotherapy. Mouse survivorship increased from 25% post-DXR treatment to over 80% with Dexra pretreatment. These data demonstrate that Dexra provides acute ovarian protection from DXR toxicity, improving reproductive health in a mouse model, suggesting this clinically available drug may provide ovarian protection for cancer patients.

## Introduction

It is estimated that approximately 1 in 285 children and adolescents will be diagnosed with cancer before the age of 20 [1]. Recent advances in cancer diagnosis and therapy have increased treatment efficacy, resulting in a greater number of young cancer patients surviving to adulthood [2]. Improved cancer survivorship has driven the need to diminish the long-term morbidities of cancer therapy, including significant risk for primary ovarian insufficiency in female patients, and resultant disruption of endocrine balance and infertility[3]. Fertility preservation is now considered an essential component of cancer care to improve the quality-of-life post-cancer treatment. Current options to preserve fertility in reproductive-age women prior to cancer treatment include cryopreservation of embryos and oocytes, which do not preserve endocrine function, as well as ovarian cortical tissue cryopreservation, a procedure experimental in the U.S. [4]. Fertility preservation for pre-pubertal and adolescent girls presents a significant challenge as ovarian tissue cryopreservation is currently the only potential treatment for reproductively-immature girls, but even this invasive approach carries the risk of re-introducing cancer and raises complicated ethical ramifications [5]. Other promising therapies that may eventually be applied to pre-pubertal girls, including *in vitro* follicle maturation, are not yet clinically approved [6]. Thus, there is a demand to develop new strategies to improve fertility preservation in pre-pubertal and adolescent girls.

Understanding the biological mechanisms of ovarian injury caused by chemotherapy and its downstream consequences has become vital to developing new ovarian preservation strategies. Chemotherapy drugs, including doxorubicin (DXR), can cause toxicity in both primordial follicles and growing ovarian follicles, triggering follicular apoptosis and demise of oocytes [7–12]. DXR is an anthracyline used alone or in combination with other drugs to treat approximately 50% of all cancers, including soft and solid tumors and lymphomas [1,13–16]. The widespread use of DXR necessitates strategies to counteract undesirable ovarian toxicity. At the cellular level, DXR can accumulate in both the nucleus and mitochondria of target cells, causing DNA damage and oxidative stress [17]. DXR induces double-strand DNA (dsDNA) breaks by intercalating into DNA, thereby inhibiting the resealing action of topoisomerase II (TOP II) during normal DNA replication [17–19]. One potential strategy to prevent DXR toxicity is therefore inhibiting TOP II-mediated DNA cleavage to prevent accumulation of dsDNA breaks, while allowing time for the cell to metabolize and remove DXR.

We previously characterized the time course of DXR accumulation, DNA insult and resultant apoptosis within the ovaries of adolescent female mice to provide a mechanistic framework for testing putative ovoprotective agents. The study revealed that a single injection of DXR caused cell type-specific and time-dependent toxic effects in the ovaries of female mice [12]. DNA damage was detectable in stromal cells as early as 2 hours (h) post injection, then penetrated into the granulosa cells causing concomitant dsDNA breaks by 4 h, followed by continued radial penetration into the follicles. DNA damage was followed by granulosa cell apoptosis and oocyte DNA damage by 12 h post-injection. As both the stromal and granulosa cells offer support to oocyte function, an agent that would protect the ovary from DXR-induced toxicity needs to prevent acute insult in these supporting cells as well as oocytes.

Dexrazoxane (Dexra), an iron-chelating EDTA derivative, is the only drug clinically used to diminish the off-target effects of DXR in cardiac and skin tissue without significantly limiting the effectiveness of treating the cancer [20]. Dexra mitigates oxidative stress by chelating iron (disrupting DXR-iron binding) and catalytically inhibiting TOPII, thus preventing DXR-induced dsDNA breaks [21,22]. Cardiotoxicity is one of the most serious complications arising from DXR treatment and therefore has been the focus of much work to prevent DXR toxicity. In patients at-risk for cardiac complications, Dexra is administered prior to DXR treatment at a 10:1 milligram (mg) Dexra:DXR dose to provide cardiac protection [20].

In a proof-of-concept study using immortalized KK-15 mouse granulosa cells, we demonstrated that Dexra protects granulosa cells from DXR-induced DNA damage and cytotoxicity *in vitro* in a manner consistent with inhibiting TOPII DNA cleavage, rather than oxidative stress [23]. Furthermore, Dexra treatment of *in vitro-*cultured mouse and marmoset ovaries prior to DXR administration reduced the number of dsDNA breaks, and rescued primary granulosa cell viability [23,24]. The data demonstrated the potential for Dexra to be an effective ovoprotective agent against DXR toxicity by blocking the initial insult as well as subsequent cellular demise.

In the present study, we utilized a mouse model to determine whether Dexra offers both acute and long-term ovarian protection from DXR injury *in vivo*. Adolescent female mice were pretreated with Dexra prior to DXR administration at a 1:1 mg dose ratio to assess whether Dexra protects from acute DXR-induced DNA damage and follicular apoptosis within the first 24 h of drug administration. To determine whether Dexra also improves long-term reproductive health following DXR treatment, adolescent female mice were administered either a 1:1 or 10:1 mg Dexra:DXR dose ratio and number of litters, litter sizes and pup weights were quantified as mice were continually mated throughout their reproductive lifespan. This *in vivo* mouse study demonstrates the potential to utilize Dexra as a cost-effective ovoprotective agent, providing a mechanistic approach to preserving ovarian function as well as fertility and fecundity for young female cancer patients.

## Materials and Methods

### Chemicals

Doxorubicin hydrochloride (2 mg/mL in 0.9% sodium chloride) was from Teva Parenteral Medicines (Irvine, CA). Dexrazoxane hydrochloride (powder) from Pharmacia & Upjohn (New York, NY) was solubilized as 80 mM in 0.167 M sodium lactate immediately prior to use, and diluted to the final concentration prior to injection.

### Mice

Animal studies were conducted in accordance with the Guide for the Care and Use of Laboratory Animals and the Animal Welfare Act. The Institutional Animal Care and Use Committee of the School of Medicine and Public Health at the University of Wisconsin-Madison approved all protocols and procedures prior to implementation. All surgery was performed under Ketamine and isofluorane anesthesia. Female CD-1 mice were purchased at 3 weeks of age from Charles River Laboratories (Wilmington, MA) and allowed to acclimate to the laboratory environment for one week prior to the start of an experiment under the supervision and care of the animal facility staff. At 4 weeks of age, the adolescent mice were injected with Dexra or vehicle control (0.0167 M lactate in saline) via intraperitoneal injection using ≤ 200 μL/injection 1 hour prior to DXR injection. DXR or vehicle (saline) was subsequently administered via intraperitoneal injection.

#### Acute treatment

At 4 weeks of age, mice were treated with 1) Vehicle for Dexra + Vehicle for DXR, 2) Vehicle for Dexra + 20 mg/kg DXR, 3) 20 mg/kg Dexra + Vehicle for DXR, or 4) 20 mg/kg Dexra + 20 mg/kg DXR; doses were calculated based on the average weight of a 4-week-old CD-1 mouse. The 20 mg/kg DXR dose represents twice the maximum human equivalent DXR dose and was chosen in order to engage ample acute DXR toxicity [25]. The 20 mg/kg Dexra dose represents a 1:1 Dexra/DXR mg ratio, providing a significant dose reduction from that used in cardioprotection to limit potential side effects of Dexra. The chosen Dexra dose was based on our previous *in vitro* study demonstrating a 2 μM Dexra dose, 100-folds lower than that used in *in vitro* cardiac protection studies, preserved granulosa cell viability against DXR [23]. Animals were euthanized with CO_2_ followed by cervical dislocation and ovaries removed surgically 0, 2, 4, 10, 12 or 24 h after the second injection. Experiments were carried out in 4 biological replicates in which 3 mice were treated per drug group and harvested for each time point per biological replicate; in sum, n = 12 animals per treatment were totaled across all replicates. Ovaries were placed in 2 mL phosphate buffered saline, pH 7.4, and cleared of fat and attached bursa. For each ovarian pair, one was fixed in 10% formalin and processed for TUNEL assay, and the second was processed for a neutral comet assay. Separate mice were treated to provide ovaries utilized for protein extraction followed by Western blot analysis as previously described [12,23].

#### Breeding trial

Female CD-1 mice were housed in Innovive system cages (Innovive, San Diego, CA) from 3 weeks until 8 months of age. At 4 weeks of age, mice were treated with: 1) Vehicle for Dexra + Vehicle for DXR, 2) Vehicle for Dexra + 10mg/kg DXR, 3) 10mg/kg Dexra (1:1 mg ratio) + 10mg/kg DXR, 4) 100 mg/kg Dexra (10:1 mg ratio) + 10mg/kg DXR, 5) 10mg/kg Dexra (1:1 mg ratio) + Vehicle for DXR, or 6) 100mg/kg Dexra (10:1 mg ratio) + Vehicle for DXR. DXR was administered at 10 mg/kg body weight (a human equivalent dose of 30mg/m^2^) to minimize long-term cardiotoxicity. Dexra dose is expressed as a ratio to DXR dose throughout the manuscript. Dexra was administered at either a 1:1 mg ratio (labeled as Dexra_1_:DXR_1_, groups 3 above) or 10:1 mg ratio (labeled as Dexra_10_:DXR_1_, group 4 above, currently used in cardioprotective protocols) to DXR as indicated. Dexra control-treated animals (groups 5 and 6, above) are labeled as Dexra_C_ (Dexra_C1_ and Dexra_C10_ respectively) throughout the manuscript. At 6 weeks of age and prior to breeding, animals were treated for two weeks with drinking water medicated with enrofloxacin (Baytril; 22.7 mg/ml) at a calculated dose of 5 mg/kg (0.5 mL/300 mL ddH_2_O bottle; Bayer HealthCare LLC, Kansas) as a prophylactic to mitigate the side effects of a compromised immune system brought on by DXR treatment. At 8 weeks of age, females were moved to breeder cages where two females were paired with one male. Females were continuously mated from 8 weeks of age to 8 months of age or until 6 litters were achieved. Males were rotated following each breeding round to minimize any potential male-specific infertility effect. Animals within the breeder cage were fed a maintenance chow diet with protein: 24%; Fat: 4%; Fiber: 4.5% (Harlan Laboratories #8604, Indianapolis, IN) as well as irradiated sunflower seeds. Bi-weekly assessment of animal health was conducted, and additional nutritive support via DietGel^®^ (ClearH_2_O^®^, Portland, ME) and sunflower seeds was given to females having difficulty maintaining body condition. Females remained within the breeder cage until they showed visual or palpable signs of pregnancy, at which point they were separated and maintained on a breeder irradiated diet, Harlan Laboratories #2919 (Protein: 19%; Fat: 9%; Fiber: 5%) until parturition. The health of the breeding mice was monitored at least three times daily when the mice were near parturition.

Following delivery, pups were separated and the females were returned to the breeder cage within 24 h post-partum. The pups were counted, weighed, and euthanized on post-natal day 1 (PND1). At 8 months of age, the now non-pregnant dams were weighed, anesthetized with isoflurane (confirmed with limb pinch) and sacrificed via terminal blood draw followed by cervical dislocation. A terminal blood draw was carried out for future studies. Ovaries were removed from each female and weighed. Mice that did not survive to breeding age or that displayed signs of deteriorating health were removed from the breeding trial to minimize any suffering. The breeding trial was carried out in 4 replicates, with 3–6 mice per group per replicate, where the total number of female mice in each group at the start of breeding was 16 control, 16 DXR, 21 Dexra_1_:DXR_1_, 16 Dexra_10_:DXR_1_, 12 Dexra_C1_, and 12 Dexra_C10_ across all 4 replicates. Data for survival analysis, pup weights, and litter sizes were included for analysis at the intervals for which the dam was present in the trial. Infertility index was conducted on mice that gave birth at each mating round and ovarian weight analysis was conducted at 8 months.

Animals were monitored by frequent visual and physical examinations of each animal with regard to food intake, normal activity, movement, grooming, failure to thrive, weight loss, and any signs of discomfort or infection. The University of Wisconsin-Madison Research Animal Resources Center Veterinarian Staff was contacted regarding any animal health concerns; and sick animals were monitored more closely. Dams that exhibited signs of dystocia were supplied thermal support by externally warming the cage via heated surface or ceramic heat lamp, DietGel^®^ nutritional support (ClearH_2_O^®^, Portland, ME) was provided, mice were hydrated subcutaneously with 1 to 1.5 ml saline (based on adult mouse weight) and were monitored closely. Analgesics agents (subcutaneous Meloxicam at a dose of 1–2 mg/kg per day) were given to minimize animal suffering and distress. Many of the dams with dystocia did not respond to treatment, and were euthanized via CO_2_ followed by cervical dislocation to avoid further complications. Cesarean section was not utilized due to the risks imposed on pregnant dams, as cesarean section is often a terminal procedure in mouse dams. DXR-treated animals exhibited signs of poorer health. These animals were provided additional DietGel^®^ nutritional support (ClearH_2_O^®^, Portland, ME) and monitored closely. Animals were euthanized, via CO_2_ followed by cervical dislocation, if they did not respond to palliative treatment had a poor body condition score, ruffed fur/coat, hunched posture, were rough, lethargic, and/or moribund [26]. Necropsy assessment of mice removed from the trial revealed that these animals developed pregnancy-related complications such as retained fetuses and uterine infection as well as peritonitis, and bone marrow myeloid hyperplasia; non-pregnancy related causes. Necropsy was not performed on all DXR-treated animals, however, gross anatomical examination of other organs during necropsy including the heart, lungs, liver, kidneys, intestine, and pancreas appeared normal for pregnancy. Overall, the degree of animal compromise was mitigated by the substantial nutritional and antibiotic support that was added in response to noting the challenges associated with DXR treatment in the first trial.

All efforts were taken to decrease the number of animals used. Data from vehicle control and DXR-only treated animals in this manuscript have been reported previously in a study examining bortezomib protection against DXR toxicity [27]. The initial study design comprised eight treatment groups, including control-, bortezomib- and dexrazoxane-treated mice (S1 Table). Animals in all of these groups were treated at the same time and side-by-side, but were maintained independently in separate cages, and were handled discretely across all experimental replicates. In this design, the control and DXR-only treated animals did not need to be duplicated, and thereby decreased overall animal usage by ~ 25%.

### Neutral Comet Assay

Ovarian tissue was separated into cell populations, one enriched for granulosa cells and oocytes and the second for stroma and theca cells, as previously described [12,23]. Briefly, antral follicles were gently punctured to release granulosa cells and oocytes, followed by incubation with 0.1% proteinase K to digest the zona pellucida. Residual tissue enriched for stroma and theca cells was treated with collagenase IV (0.25% in PBS, pH 7.4) followed by passage through a 23-gauge needle to disperse cells [12,23]. Cells were mixed with equal volume 1% low melting point agarose in PBS at 37°C, lysed in-gel, and electrophoresed. DNA was stained with propidium iodide and imaged [12,23]. Images were collected from blinded samples on an Olympus microscope using a 20X objective and SPOT Plus software. For granulosa and stroma/theca cells, >100 cells per time point per mouse were imaged, while 50 oocytes were imaged per time point per mouse. Imaged comets were scored using CometScore software (TriTek Corporation) as described previously [12,23]. Experimental data were normalized to control data so that data from separate experiments could be pooled.

### Lysate preparation and Western blots

Nucleus and cytoplasm-enriched protein lysate fractions were isolated from homogenized mouse ovaries and protein concentration was quantified using the Biorad DC Protein Assay (Bio-Rad, Hercules, CA) according to manufacturer’s instructions. Protein fractions (10–20 μg per lane) were prepared in Laemmli Sample Buffer, heated 10 min at 65°C, size-separated by SDS-PAGE, transferred to PVDF-Fl (Millipore, Billerica, MA) membranes, and pre-blocked in TBS-T (0.05% Tween-20) + 5% BSA for 1 h at room temperature as previously described [23]. Membranes were incubated overnight at 4°C in TBS-T + 5% BSA containing polyclonal rabbit anti-S139-phosphorylated **γ**H2AFX (1:500, catalogue number ab11174, Abcam, Cambridge, MA) and monoclonal mouse anti-β actin (1:10,000, catalogue number A5316, Sigma, St. Louis, MO) as a loading control. Blots were washed with TBS-T then incubated simultaneously for 1 h at room temperature with donkey anti-rabbit Alexa 680 (1:15,000 in TBS-T; catalogue number A10043, Molecular Probes, Grand Island, NY) and donkey anti-mouse IRDye 800 (1:15,000 in TBS-T; catalogue number 926–32212, LiCor, Lincoln, Nebraska). Blots were washed with TBS-T, dried, scanned and quantified using the LiCor Odyssey System (University of Wisconsin-Small Molecule Screening Facility) and Odyssey software.

### TUNEL Assay

Fixed ovarian sections (5 μm) were stained using the ApopTag Plus Fluorescein In Situ Apoptosis Detection Kit, as described previously (Millipore) [12]. Slides were counterstained with 0.5 μg/mL propidium iodide and imaged on a Nikon A1 confocal laser microscope with motorized stage (WNPRC for Biological Imaging, UW-Madison) using sequential laser scanning. Follicle types were identified using standard morphology and size ranges [28]. The mean TUNEL-positive index was calculated as the (number of TUNEL-positive follicles)/(total follicle count) for each follicle type based on 4 ovaries/time point across 4 replicates. Follicles lacking a visible oocyte were not scored. Growing follicles were considered apoptotic if they had ≥4 TUNEL-positive granulosa cells, respectively, while primordial follicles were considered apoptotic if they had ≥1 TUNEL-positive granulosa cells [12,28].

### Statistics

Analyses of the data and graphs were completed using OriginLab. Bonferroni post-hoc means comparison was used throughout with the exception of the Western analysis, which did not meet the stringency for Bonferroni with an n = 3, but was significant with Tukey means comparison. Two-way ANOVA was used in each example of multi-parameter experiments (time and treatment), where one-way ANOVA was used for single-parameter experiments (different treatments all assessed as a single endpoint). Data presented include means for each experimental group and standard error (SE). To assess the difference in infertility index, an analysis of covariance (ANCOVA) test was performed, where test data were limited to births 3–6 to avoid the lack of variance and ceiling effect observed with outcomes for births 1–2. Analysis of covariance showed homogeneity of slopes (p>0.05) for outcomes of treatments across births. Consequently, a two-way ANOVA was performed after arcsine transformation, followed by separate one-way ANOVA with Bonferroni-adjusted posthoc means comparisons. For survival analysis, a log-rank test was used to assess difference between groups. For each measure, the p-value was set as p<0.05.

## Results

### Neutral comet assay demonstrates Dexra shielded ovarian cells from DXR-induced DNA damage

**Fig 1:**
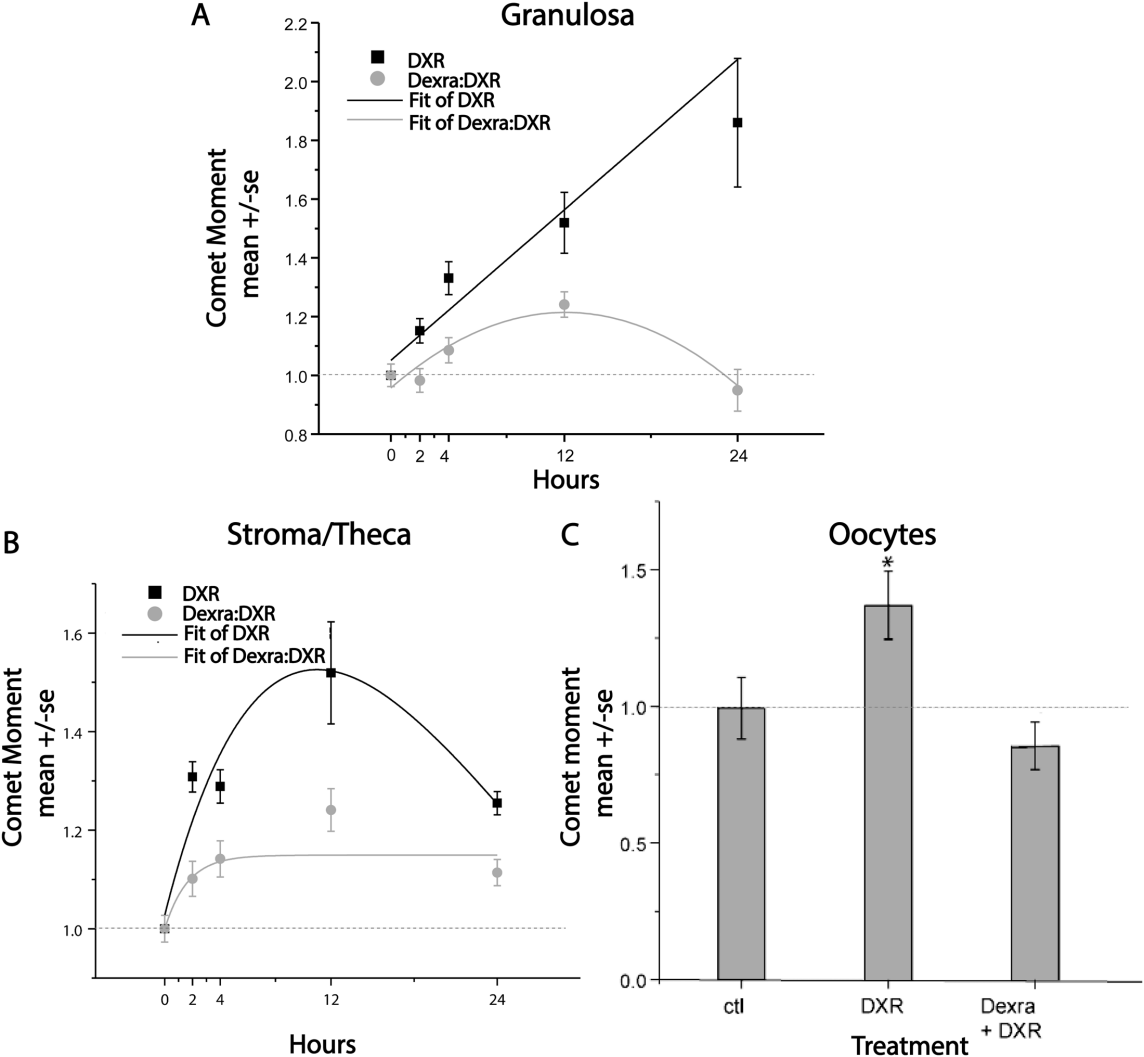
Dexra prevents DXR-induced dsDNA breaks in ovarian cells. Neutral comet assay was performed on the indicated cell types. Panel ***A*** quantifies dsDNA breaks in granulosa-enriched cell populations at time points from 0–24 h post-DXR injection. Panel ***B*** quantifies dsDNA breaks in stromal/thecal-enriched cell populations at time points from 0–24 h post-DXR injection. Panel ***C*** bar graph quantifies dsDNA breaks in oocytes at 24 h post-DXR injection. n = 3 mice/group/point, per replicate, 4 replicates. * p< 0.05, one-way ANOVA. The control and DXR-only treatment groups adapted from [27].

To determine whether Dexra protects against DXR-induced DNA damage, dsDNA breaks were quantified as the Olive Moment (OM) following neutral comet assay lysis and electrophoresis of ovarian cells isolated from 4 week-old (adolescent) female mice 0–24 hours post- *in vivo* treatment with vehicle control, 20 mg/kg DXR, or 20 mg/kg Dexra + 20 mg/kg DXR. As illustrated in Fig 1A, DXR induced a time-dependent increase in dsDNA breaks in granulosa cells, peaking at 1.8-times greater than control by 24 h. This increase was attenuated in Dexra:DXR treated mice to levels within 10% of control values in granulosa cells from ovaries removed at all tested time points through 24 h after injection with DXR (Fig 1A). Similarly, Dexra decreased the DXR-induced ~1.5x fold increase in dsDNA breaks in stromal/thecal-enriched cell fractions to levels within ~12% of control values through 24 h post-DXR injection (Fig 1B). Oocytes exhibited ~1.4x increase in the quantity of dsDNA breaks 24 h post-DXR injection when compared to the vehicle control (Fig 1C). Dexra pretreatment restrained dsDNA breaks in oocytes to those values found in controls. dsDNA breaks were quantified in oocytes only at 24 h time points, as our previous studies showed DXR-induced dsDNA breaks were not detected in oocytes before 12 hours [12]. These data demonstrate Dexra shielded the ovary from DXR-induced DNA damage in adolescent female mice throughout the entire acute insult period and across all ovarian cell types. Dexra-mediated inhibition of DNA damage lasted for 24 hours, well beyond the time of DXR blood clearance in human patients (15–30 minutes) [25], indicating Dexra has the potential to mitigate DXR-induced DNA damage until it is cleared from the circulation.

### Dexra pretreatment reduced DXR-induced *γH2FAX* phosphorylation in mouse ovaries

**Fig 2:**
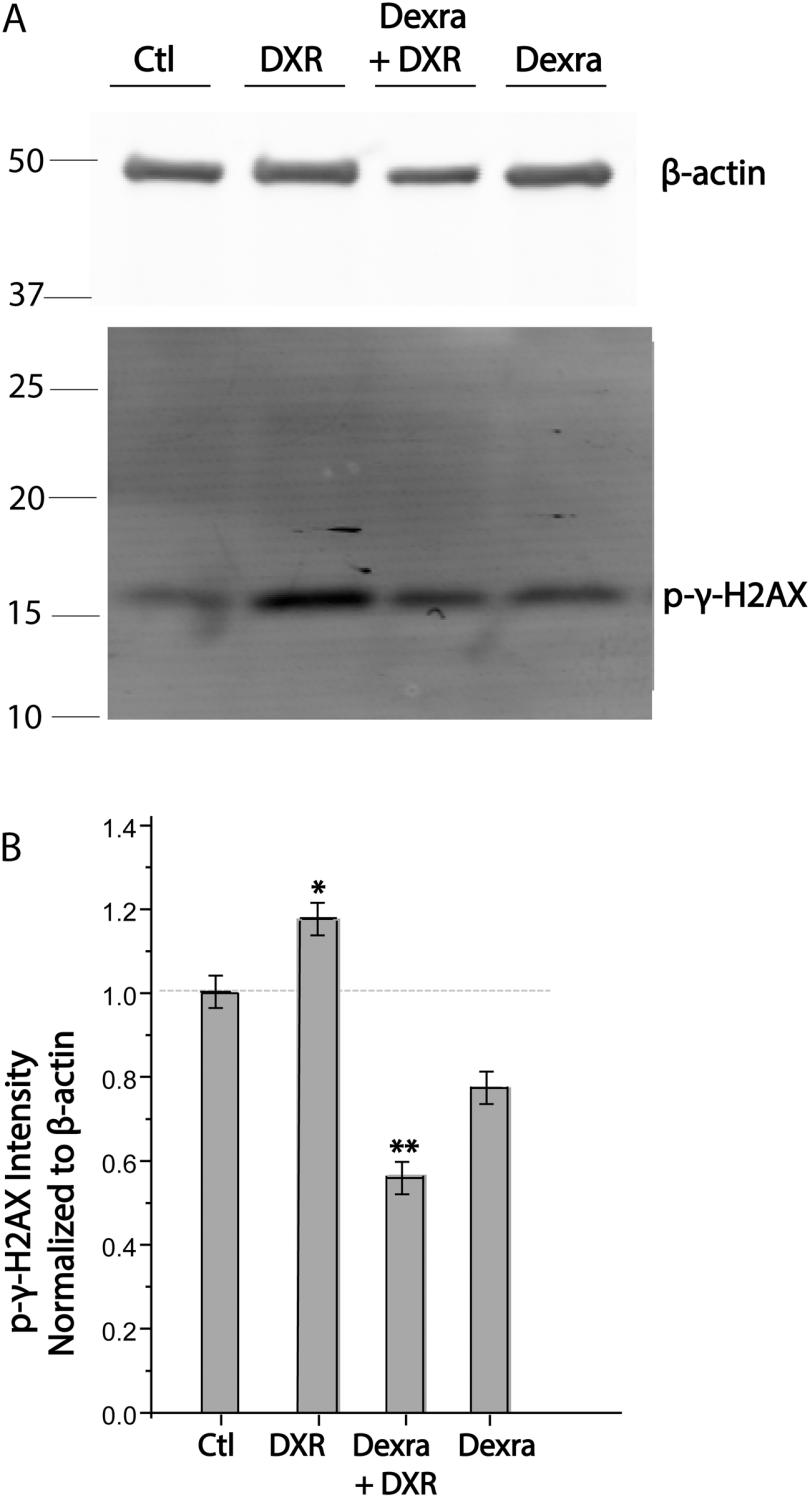
γH2AFX activation within ovarian cells is reduced in response to Dexra pretreatment *A*. Western blot of ovarian lysates at 6 h post DXR-injection probed with antibodies to **γ**H2AFX (17 kDa) or β-actin (42 kDa); ***B*.** Quantification of (*A*); values normalized to β-actin. n = 3 blots/quantification, error bars indicate the SE of the mean, * p< 0.05, **p<0.001, one-way ANOVA, Tukey means comparison. The control and DXR-only treatment groups adapted from [27].

Phosphorylation of **γ**H2AFX reports the cellular response to dsDNA breaks in eukaryotic cells [29], and was quantified by Western blot analysis of ovarian lysates from all treatment groups to test whether Dexra prevents DXR-induced **γ**H2AFX activation. **γ**H2AFX phosphorylation increased by 20% (p<0.05, one-way ANOVA) in ovaries from DXR-treated mice compared to vehicle control. In contrast, Dexra administered prior to DXR diminished ovarian **γ**H2AFX phosphorylation below vehicle control values (a 55% decrease from DXR treatment, p<0.001, one-way ANOVA, Fig 2A and 2B, and a 40% decrease from controls). These data not only demonstrate Dexra diminishing DXR-induced **γ**H2AFX activation in ovaries of adolescent female mice (Fig 2A and 2B), but also preventing DXR-induced dsDNA breaks (Fig 1).

### DXR-induced follicle apoptosis mitigated by Dexra pretreatment

**Fig 3:**
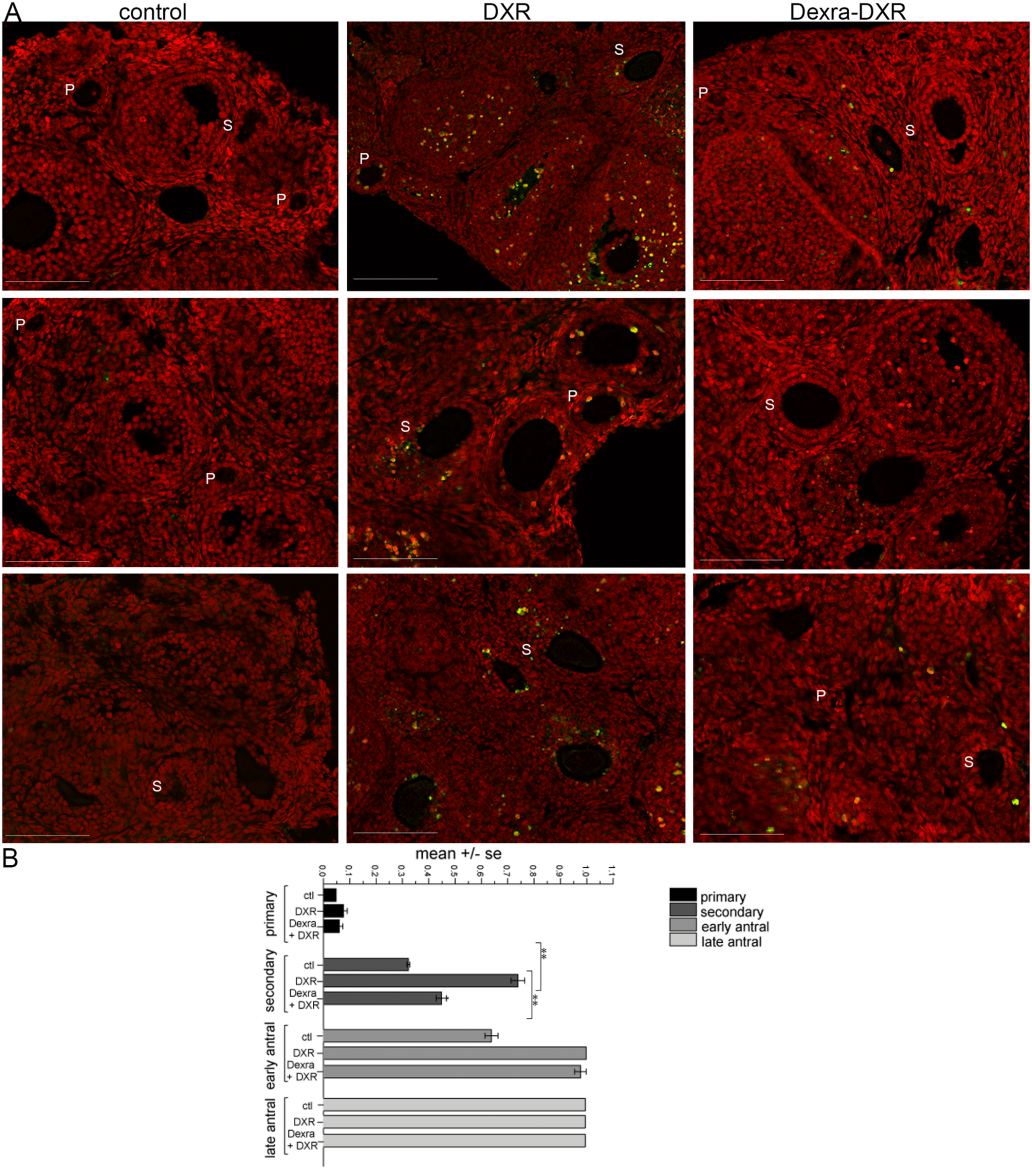
Apoptosis is reduced in ovarian follicles of Dexra pretreated mice in comparison to DXR-alone treated mice. Apoptosis was detected by TUNEL assay at 12 h after DXR injection. ***A*.** Representative micrographs of digitally magnified selected areas of ovaries from three different mice for each treatment condition are shown. Ovarian sections were stained with TUNEL (green) or PI (red, nuclei); Insets, scale bar = 100 μm for all images. All images were adjusted +30 brightness and +15 contrast to enhance visibility in print. ***B*.** Corresponding quantification of the apoptotic index in follicles. TUNEL-positive index across the different follicle types is calculated as the fraction of TUNEL-positive follicles vs. total follicle count for each follicular class. S = secondary follicles, P = primary follicles, n = 3 mice per treatment carried out in 4 replicates, error bars indicate the SE of the mean, **p<0.001, one-way ANOVA, Bonferroni means comparison. The control and DXR-only treatment groups adapted from [27].

To test the hypothesis that Dexra abrogates DXR-induced follicle apoptosis, the mean apoptotic index was calculated from images of TUNEL assay staining in ovarian sections at 12 h post-treatment (Fig 3). Injection with DXR increased the mean apoptotic index by 25% in primary follicles and doubled the apoptotic index in secondary follicles (p>0.05, p<0.001 respectively, one-way ANOVA) compared to control (Fig 3A, micrographs and Fig 3B, quantification). Consistent with our previous report [12], primordial follicles did not exhibit DXR-induced apoptosis at the 12 h time point (data not quantified). In ovaries from mice treated with Dexra prior to DXR, the degree of secondary follicle apoptosis was similar to that of control, and importantly, was 30% lower than that observed in DXR-treated ovaries (p<0.001, one-way ANOVA, Fig 3B quantification). In all treatment groups, antral follicles exhibited TUNEL-positive granulosa cells, with no significant changes between groups. Consistent with a previous study, antral follicles exhibited a relatively high incidence of baseline apoptosis [27]. S1 Fig shows the entire ovarian section for the corresponding zoomed images in Fig 3A. Mice treated with Dexra alone exhibited minimal TUNEL-positive follicles (data not quantified). These data demonstrate Dexra ameliorated the demise of secondary follicles following DXR treatment.

### Dexra pretreatment prolonged the fertility window following DXR treatment

**Fig 4:**
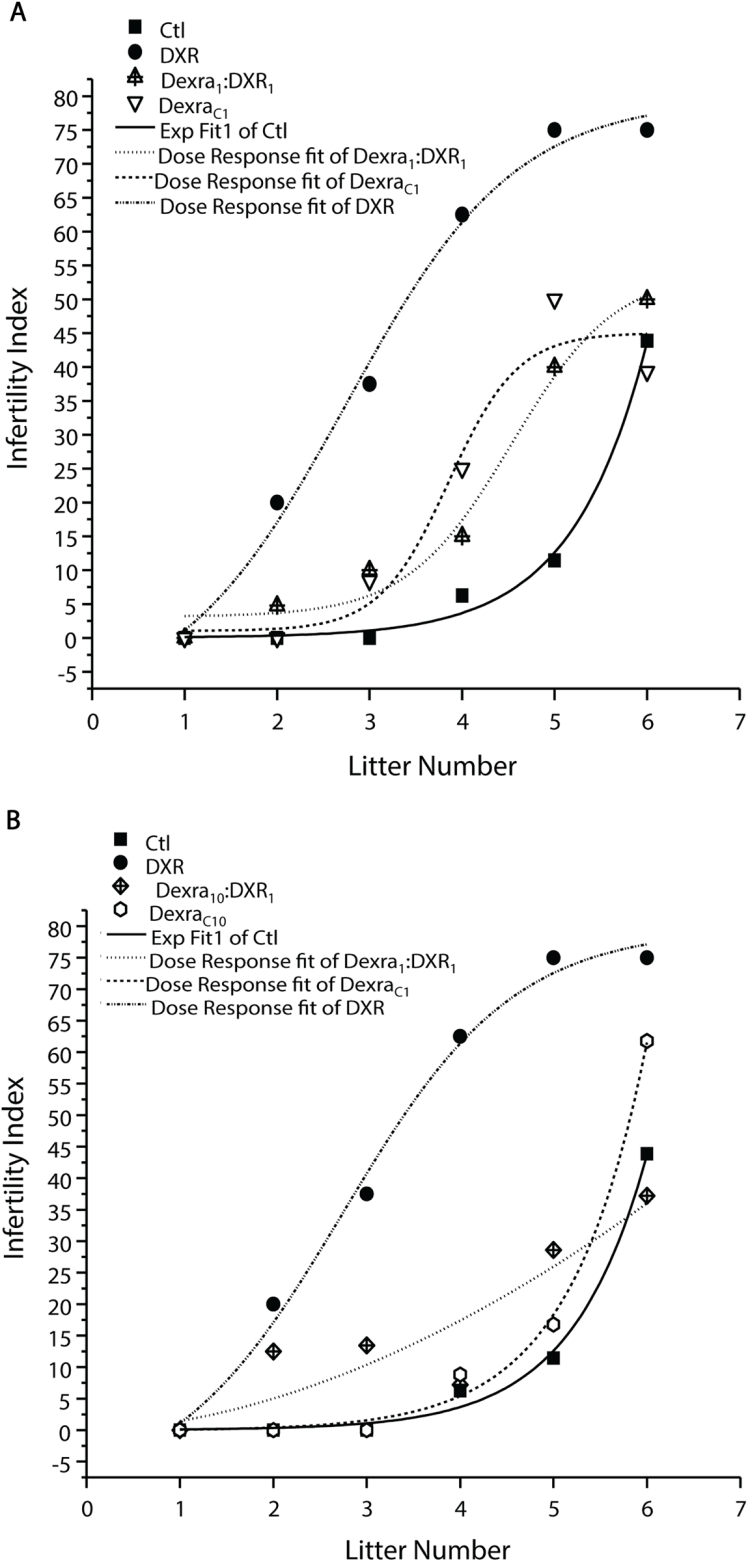
Dexra pretreatment prevents DXR-induced infertility of mice over time. An “infertility index” was calculated as the percentage of surviving mice that failed to achieve pregnancy and deliver at 30-day intervals through 8 months of age. Litter number was designated as the litter that should have been achieved by the 30-day interval predicted by a 21-day gestation length and equivalent observation in control animals. Symbols correspond to different treatment groups as indicated. ***A*.** Graph of infertility index of the Dexra_1_:DXR_1_ (1:1 mg ratio dose), ***B*.** Graph of infertility index of the Dexra_10_:DXR_1_ (10:1 mg ratio dose). (p<0.05, two-way ANOVA, Bonferroni means comparison). n = 18 for Control (vehicle control), 4 for DXR, 15 for Dexra_1_:DXR_1_ (1:1 mg ratio dose), 11 for Dexra_10_:DXR_1_ (10:1 mg ratio dose), 12 for Dexra_C1_, and 12 for Dexra_C10_, where n represents the number of mice that survived to the end of the study. The control and DXR-only treatment groups adapted from [27].

The breeding study was conducted to test the hypothesis that Dexra treatment preserved long-term mice fecundity against DXR toxicity. In addition to the low Dexra_1_:DXR_1_ dose ratio (1:1 mg ratio) utilized for the acute studies, we added a higher Dexra_10_:DXR_1_ dose ratio (10:1 mg ratio) to represent the current clinical practice for cardiac and skin protection. To test the hypothesis that Dexra maintains fertility in the face of DXR ovarian insult, an 'infertility index' was calculated and defined as the percentage of surviving females that fail to deliver after the subsequent mating round. The ‘‘infertility index” is plotted vs. litter number in Fig 4. The infertility index of DXR-treated mice revealed a significant loss in fertility by the 3^rd^ gestation following treatment, reaching a plateau with 75% of animals failing to deliver a 5^th^ litter (p<0.001, Bonferonni-corrected posthoc means comparison over the linear range, 3^rd^ to 6^th^ gestation, Fig 4A and 4B). Dexra:DXR treated animals, at either 1:1 (Dexra_1_:DXR_1_) or 10:1 (Dexra_10_:DXR_1_) doses, demonstrated comparable fertility to controls, as well as to Dexra only-treated mice. Dexra only-treated animals did not differ from control (p>0.05, two-way ANOVA over the linear range); both groups exhibited age-related declines, reaching ~45% infertility at litter 6. These data demonstrate that Dexra pretreatment at either dose prior to DXR improved the remaining fertility window over the reproductive lifespan of the mice compared with DXR alone.

### DXR-induced reduction in pup birth weight was improved by Dexra pretreatment

**Fig 5:**
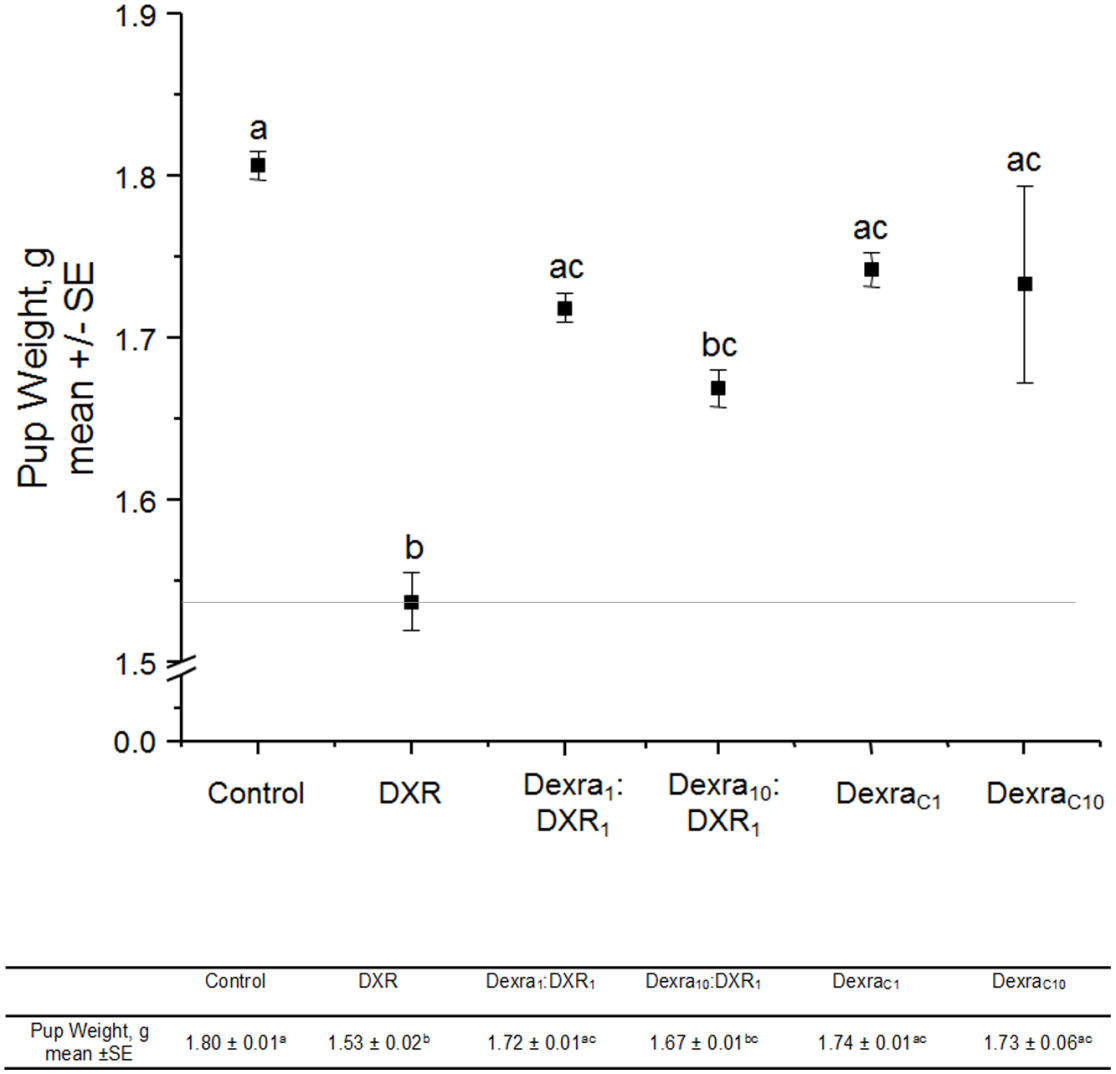
Dexra pretreatment prevents DXR-induced decrease in pup weight. Weight measured in grams (g) on PND1 and represented as mean weight for each treatment. Error bars indicate the SE of the mean pup weight. Letters above data points represent groups that significantly differ from one another, one-way ANOVA with Bonferroni means comparison, n = 939 pups for Control (vehicle control), 175 for DXR, 524 for Dexra_1_:DXR_1_ (1:1 mg ratio dose), 703 for Dexra_10_:DXR_1_ (10:1 mg ratio dose), 712 for Dexra_C1_, and 428 for Dexra_C10_. The control and DXR-only treatment groups adapted from [27].

On postnatal day one, birth weights were collected from each pup to test the hypothesis that Dexra treatment of dams maintained pup weight despite DXR insult. Pups born to DXR-treated dams had weights lower than vehicle control-treated animals, 1.53 ± 0.02 vs. 1.80 ± 0.01 g, respectively (Fig 5; p<0.05, one-way ANOVA, Bonferroni means comparison). Pups derived from dams treated with Dexra_1_:DXR_1_ had a mean birth weight of 1.72 ± 0.01 g that was not different from controls (Fig 5; p = 0.3, one-way ANOVA), while exhibiting a trend (p<0.06) toward increased mean birth weight compared to DXR alone. In contrast, pups derived from dams treated with Dexra_10_:DXR_1_ had a mean pup weight of 1.67 ± 0.01 g that was lower than control (Fig 5; p<0.05, one-way ANOVA). As the focus of this study was on low dose Dexra protection, it is worth noting that fewer animals were enrolled in the high dose Dexra protection arm. Dexra alone was well tolerated, as pups derived from Dexra_C1_ and Dexra_C10_ dams weighed an average of 1.74 and 1.73 g, respectively, and were not significantly different from pups born to vehicle control dams (Fig 5). These data demonstrate that the lower dose of Dexra (Dexra_1_:DXR_1_) improved pup birth weight in dams treated with DXR. The higher dose ratio (Dexra_10_:DXR_1_) currently used for cardioprotection, however, was less effective in preventing weight loss.

### Litter size following DXR was improved by both Dexra doses

**Fig 6:**
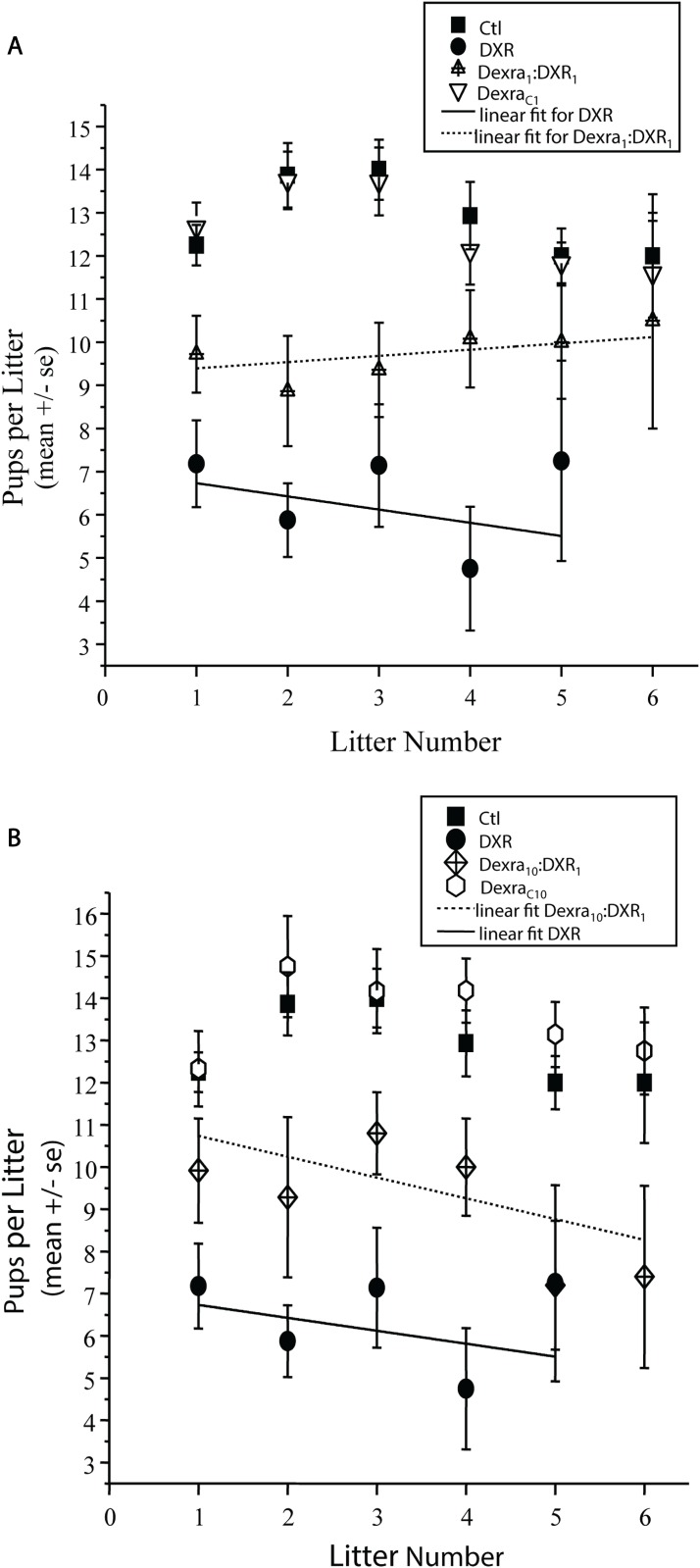
Dexra pretreatment prevents DXR-induced decrease litter sizes. Plots represent mean number of pups per litter across 6 litters. ***A*.** Graph of Dexra_1_:DXR_1_ (1:1 mg ratio dose), DXR slope = —0.31, Dexra_1_:DXR_1_ slope = 0.49. ***B*.** Graph of Dexra_10_:DXR_1_ (10:1 mg ratio dose), DXR slope = —0.31, Dexra_10_:DXR_1_ slope = 0.14. The 6^th^ litter data point of the DXR-treated animals was not represented on the graphs due to inability to calculate the SE since only one of the DXR-treated animals delivered a 6^th^ litter. The control and DXR-only treatment groups adapted from [27].

To determine whether Dexra prevented DXR-induced decrease in litter size, the numbers of pups from each dam were recorded as a function of litter number over the course of the breeding trial. Litters that showed evidence of cannibalization by the dam, while counted as a birth, were excluded from data analysis for litter size. DXR treatment reduced the average litter size from 13.0 ± 0.3 for control to 6.7 ± 0.6 pups (Fig 6; p<0.0001, two-way ANOVA). Notably, only one of the four DXR animals that survived to the end of the study delivered a 6^th^ litter. Mice treated with Dexra_1_:DXR_1_ also exhibited decreased litter size (9.5 pups ± 0.5), but the mean litter size was greater than DXR alone (p<0.001; Fig 6A). Similarly, Dexra_10_:DXR_1_ treatment improved average litter size to 10.0 ± 0.5 pups, which while still less than control (p<0.001, two-way ANOVA), was greater than DXR-treated animals (p<0.001, Fig 6B). Dexra alone did not diminish litter size in treated dams, another indication that the putative ovoprotective agent was well tolerated. The Dexra_C1_ mice had litter sizes of 13.0 ± 0.3 pups, not different from vehicle control (12.9 ± 0.3), while Dexra_C10_ litter sizes were slightly larger (p = 0.05) than vehicle control at 13.6 ± 0.4 pups. Litter sizes for both Dexra_C_ controls were greater (p<0.001) than the average DXR litter size. Litter size was independent of litter number as no significant differences were observed across litter number for any treatment (p>0.05, two-way ANOVA). These data show that both Dexra doses at either 1:1 or 10:1 mg ratio increased the litter size in comparison to DXR treatment alone, independent of litter number, demonstrating improved fecundity across all breeding rounds.

### Dexra pretreatment prevented loss of ovarian mass following DXR treatment

**Fig 7:**
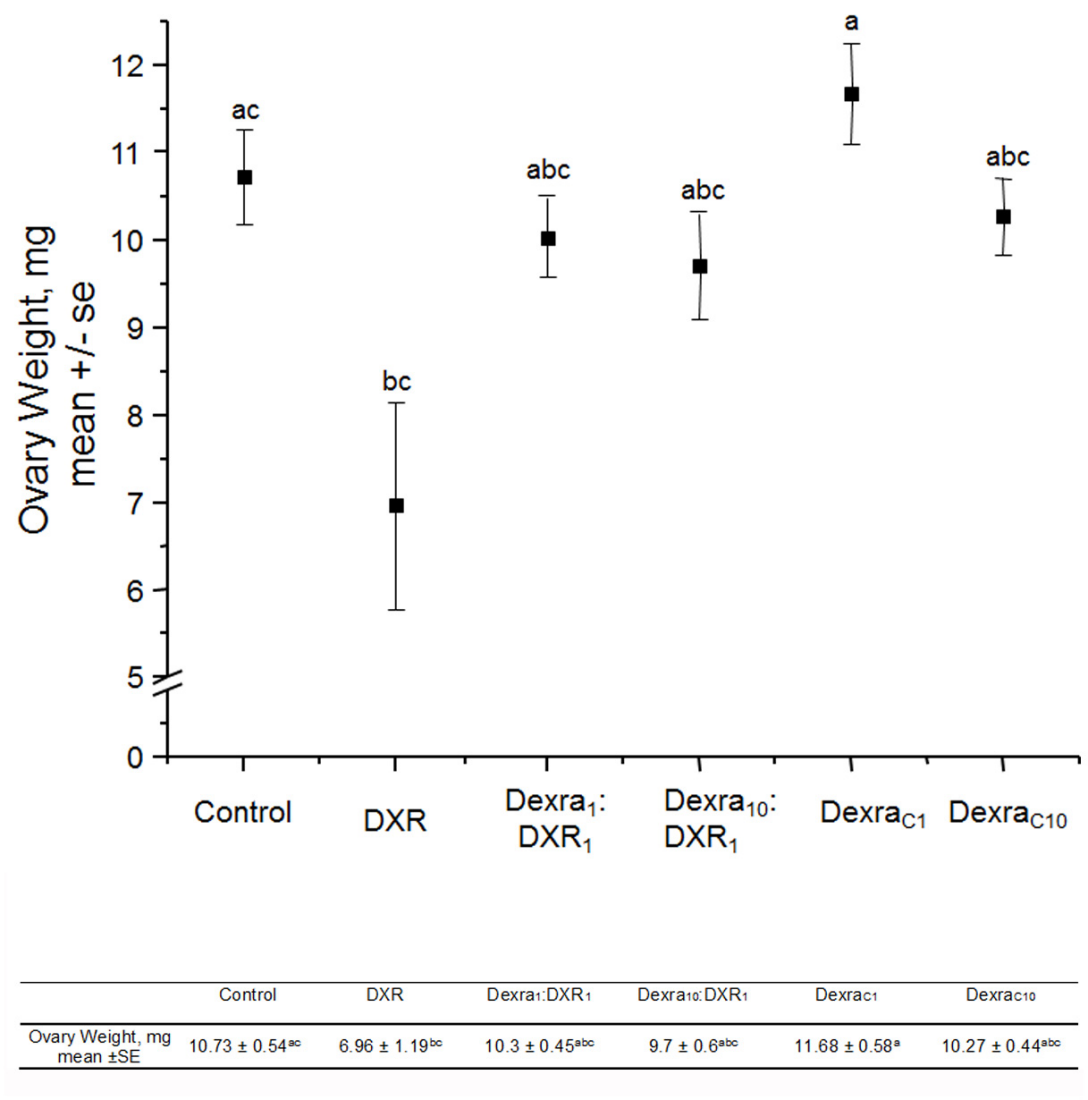
Dexra pretreatment prevents DXR-induced reduction in ovary weight. Mean weight measured in milligrams (mg). The graph plots mean ovarian weight for each treatment group at 8 months of age. Error bars indicate the SE of the mean ovary weight. Letters above data points represent groups that significantly differ from one another, one-way ANOVA with Bonferroni means comparison, n = 14 Ctl (vehicle control), 4 DXR, 17 Dexra_1_:DXR_1_ (1:1 mg ratio), 15 Dexra_10_:DXR_1_ (10:1 mg ratio), 12 Dexra_C1_, and 11 Dexra_C10_, where n represents the total number of animals in each group at the end of the study where both ovaries used for statistical analysis. The control and DXR-only treatment groups adapted from [27].

To determine whether ovarian mass decreased following DXR chemotherapy, surviving breeder mice were euthanized at 8 months of age, and ovaries were removed and weighed. The mean weights of ovaries from DXR mice were 6.96 ± 1.19 mg, a ~35% decrease (p = 0.009) from control ovarian weights (10.73 ± 0.54 mg, Fig 7). The bursa surrounding the ovaries of DXR mice were often filled with excess fluid, a phenomenon not observed in mice in any other treatment group (data not quantified). Dexra_1_:DXR_1_ treated mice had a mean ovarian weight of 10.03 ± 0.45 mg that was comparable to control, while showing a trend (p = 0.06) towards a greater ovarian weight than DXR alone. Dexra_10_:DXR_1_ treated animals exhibited intermediate ovarian weights at 9.7 ± 0.6 mg that were not different from either control or DXR-treated animals (p = 0.40). Dexra_C1_ and Dexra_C10_ mice had mean ovarian weights similar to those of control at 11.68 ± 0.58 mg and 10.27 ± 0.44 mg, respectively. These data demonstrate that the lower Dexra dose may provide better shielding from DXR-induced loss of ovarian weight.

### Dexra pretreatment improved survivorship of mice following DXR chemotherapy

**Fig 8:**
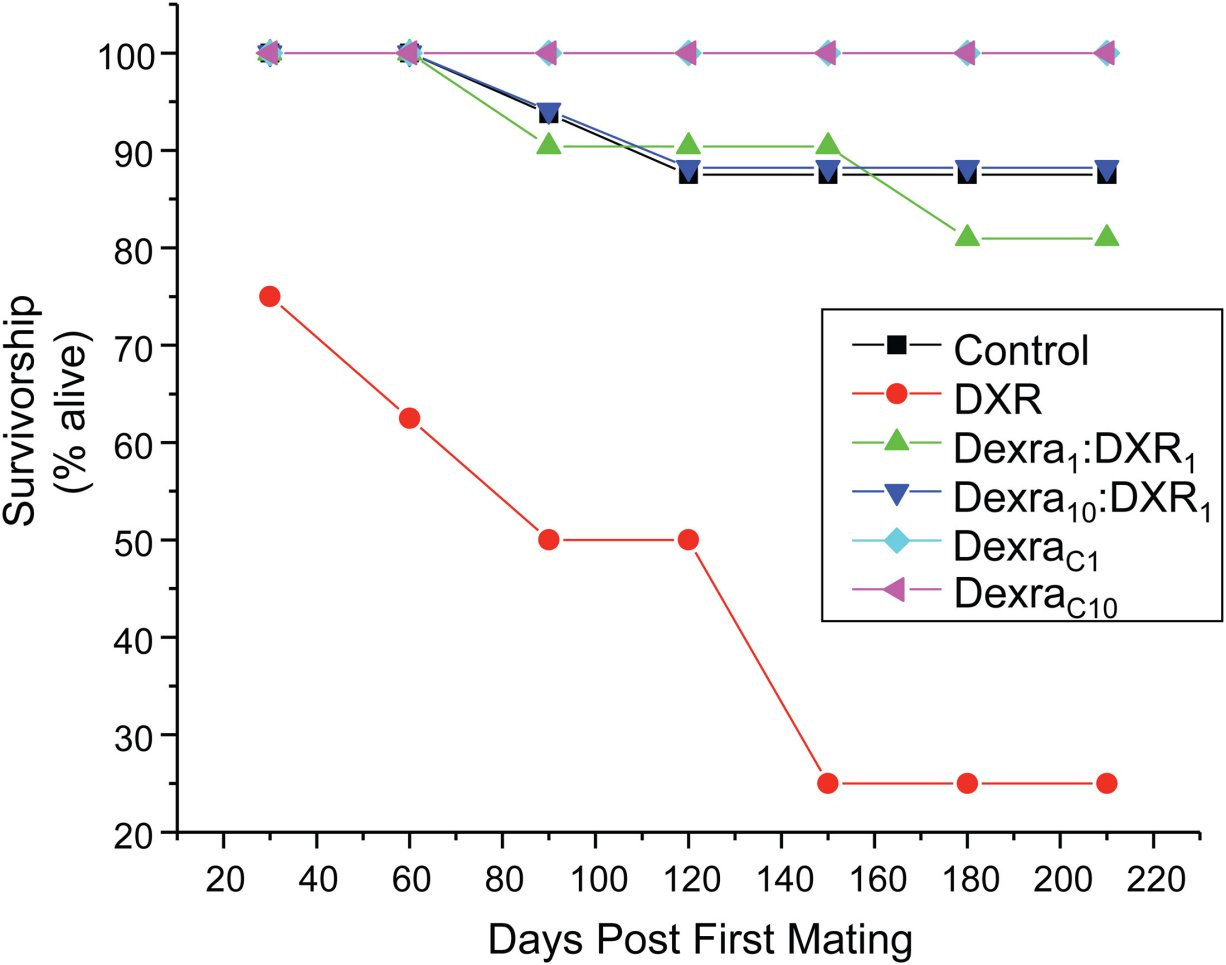
Dexra pretreatment prevents DXR-induced decrease in mice survivorship through 8 months of life. Plot represents the percentage of animals that survived as a function of days post-treatment through 8 months of age. At experiment initiation across all trials: n = 16 Ctl (vehicle control), 16 DXR, 21 Dexra_1_:DXR_1_ (1:1 mg ratio), 16 Dexra_10_:DXR_1_ (10:1 mg ratio), 12 Dexra_C1_, and 12 Dexra_C10_ mice, where n represents the total number of animals that received DXR treatment. Survival by 8 months was: n = 14 Ctl (vehicle control), 4 DXR, 17 Dexra_1_:DXR_1_ (1:1 mg ratio), 15 Dexra_10_:DXR_1_ (10:1 mg ratio), 12 Dexra_C1_, and 11 Dexra_C10_. The control and DXR-only treatment groups adapted from [27].

In human patients, DXR increases mortality due to cardiac toxicity, while Dexra administered at a 10:1 dose prior to DXR (Dexra_10_:DXR_1_) blocks DXR-related cardiotoxicity. Consistent with adverse systemic effects including cardiotoxicity, DXR reduced mouse survivorship to 25% at 30 days post initial breeding (2 months post-DXR injection, Fig 8). Survivorship continued to decline in DXR-treated mice, with a loss of 75% of DXR-treated animals by the end of the breeding trial (7 months post-DXR injection). Animals treated with DXR had a high rate of labor dystocia and often did not respond to palliative treatment (see Methods), leading to retained pups and peritonitis, contributing to morbidity. Both Dexra_1_:DXR_1_ and Dexra_10_:DXR_1_treatment groups exhibited no more than 20% survival loss over the entire course of the breeding trial (Fig 8). At 8 months of age, survivorship was 81% for Dexra_1:_:DXR_1_, 88% for Dexra_10_:DXR_1_ and 87.5% for vehicle control animals (p<0.05 for all groups when compared to DXR-treated animals, Fig 8). While survivorship advantages have been reported for high doses of Dexra (Dexra_10_:DXR_1_), these data surprisingly demonstrate that low doses of Dexra (Dexra_1_:DXR_1_) may similarly provide protection from DXR-related mortality.

## Discussion

Cryopreservation of oocytes and embryos are currently the only clinically approved options to preventing fertility loss in adult female cancer patients who undergo chemotherapeutic protocols with potential for reproductive tissue toxicity. Though successful in adult cancer patients, these techniques are neither feasible nor appropriate for pre-pubescent and young adolescent girls. Young cancer patients in particular are surviving in ever-greater numbers, creating an immediate need for novel therapies that protect the ovary from chemotherapy toxicity in children diagnosed with cancer. Drug-based ovarian protection provides a cost effective, ethically acceptable, and easy to administer alternative for fertility preservation with the potential to improve endocrine function post-chemotherapy. The present study aimed to study one drug, doxorubicin (DXR), to precisely define its toxicity and mechanisms underlying drug-based ovarian protection.

The present study demonstrates that pretreatment with Dexra shields the female adolescent mouse ovary from acute DXR insult and improves long-term fecundity in DXR-treated mice, offering a promising ovoprotective agent against one of the most common chemotherapeutic drugs used in treating childhood cancer. Dexra administered at a 1:1 mg ratio (Dexra_1_:DXR_1_) prior to DXR therapy substantially inhibited DXR-induced dsDNA breaks, **γ**H2AFX phosphorylation, and follicle apoptosis in mouse ovaries, mitigated DXR reduction in pup birth weight, and furthermore protected dams from DXR-induced mortality. Dexra therefore offers potential to provide a streamlined ovoprotective therapy that can be administered to young cancer patients in conjunction with standard chemotherapy regimens. The lower Dexra dose (Dexra_1_:DXR_1_) improved all measured outcomes following DXR treatment, and at a 10-fold less dose than that currently approved for cardioprotection, and thus promises reduced risk for Dexra-associated side effects. The high Dexra dose (Dexra_10_:DXR_1_), while also improving animal survival and protecting fertility against DXR toxicity, did not provide protection for pup and ovarian weight loss. The apparent inferior protection offered by the higher Dexra dose (which is routinely used for cardiac protection) is unclear. The mode of Dexra protection may differ between the heart and the ovary. Dexra can protect cells against DXR toxicity via two mechanisms: by catalytically inhibiting TopoII-mediated DNA cleavage and by acting as an antioxidant [20–22]. Another mechanism for Dexra-mediated protection may be down-regulation of TopoII protein [30]. Future studies are needed to determine the precise mechanism by which Dexra modulates TopoII activity and investigate molecular markers of DNA damage and repair mechanism including PARP, caspase-3, caspase-9 activation within the ovary. Dexra protects non-dividing heart cells against DXR toxicity by preventing oxidative stress [18,21]. In dividing cells such as KK15 granulosa cells, however, Dexra’s protection appears to be mediated primarily by inhibition of TopoII [23]. While a low Dexra dose (2 μM) was sufficient to prevent DXR-induced damage in KK15 mouse granulosa cells, it did not attenuate H_2_O_2_-induced oxidative stress in the granulosa cell line [23]. The finding that DXR damage to granulosa cells is likely mediated by TopoII-dependent DNA DSBs, rather than oxidative stress as in the heart, could account for the observed difference in ovarian protection exerted by low and high dose Dexra pretreatment [23]. In addition, high dose Dexra (Dexra_10_:DXR_1_) can have synergistic cytotoxic effects when combined with DXR chemotherapy [31], and it is plausible that low dose Dexra (Dexra_1_:DXR_1_) targets an appropriate therapeutic window to topoisomerases IIA. It was interesting, however, to observe that Dexra conferred better survival rates than the controls. This is likely related to Dexra-mediated antioxidant effects [32,33].

Improved mouse fertility provided by Dexra protection from DXR is consistent with a study in rats in which Dexra pretreatment provided time-dependent recovery in litter size following DXR treatment [34]. With a 20:1 Dexra:DXR dose ratio in rats, there were no differences in litter size at first parturition compared to DXR alone, but Dexra pretreatment improved litter size at second parturition [34]. As the reproductive effects of Dexra were not the primary goal of the former study, rats were only monitored through two parturitions and conclusions could not be drawn regarding long-term changes in litter size throughout the fertility window. In the present study, we followed mice continuously for 6 breeding cycles and found that Dexra administered at the low 1:1 dose ratio provided an increase in litter size compared to DXR over the entire reproductive lifespan. Previous studies have demonstrated DXR treatment reduces ovulation rate [10]; further studies will be needed to determine whether the improved litter size provided by Dexra reflects preserved ovulation rates post-DXR.

In addition to fecundity, Dexra pretreatment improved overall survivorship of mice compared to those treated with DXR alone, consistent with mitigating cardiotoxicity. While improved survivorship was expected for the 10:1 Dexra:DXR dose routinely used to prevent anthracycline-induced cardiotoxicity, the protective results were surprising for the 1:1 Dexra:DXR dose. A previous study in limited human cohorts concluded that the lower dose ratio is not sufficient to prevent DXR cardiotoxicity leading to the currently accepted 10:1 Dexra prophylactic [32,33]. Though the improved mouse survival could be due to shielding of other critical organs or species differences, revisiting the optimal dose in larger, prospective clinical studies may definitively determine whether the lower dose of Dexra is sufficient to provide cardioprotection in human patients.

The detrimental effects of DXR therapy on human reproduction are difficult to predict with certainty since DXR is often administered in combination with other chemotherapy drugs. Whether DXR toxicity is limited to the patient treated or has transgenerational consequences that negatively impact offspring has been a topic of debate. Concluding minimal transgenerational effects, Bar et al. (2003) observed that mothers previously treated with DXR as part of their pediatric cancer treatment (at a median age of 12) gave birth to babies who showed no increased risk of congenital malformations, incidence of neonatal death, nor severe morbidity [35]. In contrast, an analysis of the Childhood Cancer Survivor Study (CCSS) demonstrated patients who were previously treated with chemotherapy containing DXR were more likely to deliver lower birth weight children compared to mothers who received chemotherapy without DXR [36]. Consistent with Green et al. [36] and our present study, Bar et al. [35] demonstrated mothers treated with DXR had offspring with lower birth weights compared to matched control patients. Low birth weights have been associated with increased incidence for developing disease later in life, including cardiac and metabolic disease, and infertility [37,38]. Indeed, transgenerational complications caused by DXR treatment (5mg/kg) in mice were observed as increased neonatal death and chromosomal abnormalities in the offspring in the 4th generation (G4) of female mice following DXR treatment of generation zero (G0) dams, but not in earlier generations [39]. First developed in the 1970’s, chemotherapy has only relatively recently provided great survivor numbers, and we have not yet reached 4 human generations post-DXR. These data demonstrate the ramifications of DXR treatment in humans may be dramatically underestimated. Dexra pretreatment mitigated DXR-induced low pup birth weight in mice, suggesting Dexra pretreatment in human patients receiving DXR may similarly improve health outcomes for their future offspring. As it is widely held that reprogramming of offspring and future generations occurs *in utero* and is affected by pre-pregnancy maternal health, it is important that future studies should determine whether Dexra pretreatment prevents transgenerational abnormalities and mortality following DXR treatment in female mice. Retrospective studies on children of DXR-treated cancer survivors and subsequent generations compared to those pre-treated with Dexra could potentially determine whether the prophylactic provides benefits of protection in offspring.

The use of putative ovoprotective drugs during cancer therapy raises concerns as to whether the agent will diminish the effectiveness of the chemotherapy in treating the target cancer, i.e. reduce tumor shrinkage and survivorship. Several studies have attempted to address this safety question using a 10:1 Dexra:DXR dose, with thus far inconclusive results. While several studies have indicated Dexra does not diminish the anti-tumor effects of DXR [40–60], a study of breast cancer patients concluded the 10:1 treatment regimen reduced the tumor response rate to chemotherapy [61]. In this study, 534 patients who received either a placebo or Dexra treatment prior to DXR-containing chemotherapy were randomized to two cohorts. While Dexra decreased the tumor response rate (60% placebo compared to 48% Dexra; p = 0.019), there was no difference in survival and time to progression of the disease between the patient cohorts, [61]. A recent Cochrane review found there was no significant difference in breast cancer survival between Dexra and control groups [60]. The low 1:1 dose ratio utilized for ovarian protection in the present study may help mitigate concerns that Dexra diminishes the anti-cancer efficacy of DXR, in addition to providing chemotherapy protection to otherwise healthy organs.

Another concern with ovoprotective drugs is their long-term safety profile. A recent study in adolescents suggesting Dexra might increase risk of secondary blood cancers in children has resulted in new guidelines contraindicating the use of Dexra in young patients, who are at lower risk for cardiotoxicity [62,63]. The clinical evidence is, however, limited and controversial. The 2007 study by Tebbi et al. suggested Dexra pretreatment in adolescent patients increased risk of developing secondary tumors [62]. The study assessed the incidence of acute myeloid leukemia (AML)/myeodysplastic syndrome (MDS) and secondary tumors in 478 Hodgkin's disease patients (21 years or younger) following Dexra treatment in combination with ABVE alone (DXR, bleomycin, vincristine, and etopside) or ABVE plus cyclophosphamide. While the authors concluded increased risk for secondary cancers when using Dexra, their comparison of patients receiving comparable cumulative doses revealed three patients (out of 239) receiving Dexra plus chemotherapy developed AML/MDS, while two patients (out of 239 patients) treated with traditional chemotherapy (lacking Dexra) also developed AML/MDS [62]. It should be noted that Dexra and DXR were used in conjunction with etopside, a TopoII poison like DXR (vs. catalytic inhibitor like Dexra), which could have an additive effect in blocking TopoII DNA repair. Indeed, a 2011 study by Vrooman and colleagues assessing 553 acute lymphoblastic leukemia patients (children and adolescents) for risk of developing secondary AML when treated with 10:1 Dexra:DXR found only one patient developed AML and the 5-year cumulative incidence of secondary tumors was less than 1% in the presence of Dexra [64]. Given concerns raised by the Tebbi study, the FDA currently limits the use of Dexra to adult patients with advanced or metastatic breast cancer who have already received 300 mg/m^2^ of DXR [63]. The Vrooman study suggests the restriction on use of Dexra in adolescent patients should be reevaluated [64]. The clinical application of Dexra as an ovoprotectant at a 1/10^th^ the current clinical dose may diminish the risk for secondary tumors or altering the anti-cancer effects of DXR; if confirmed in future clinical trials, this would facilitate translation of the present study to clinical practice.

Drug-based fertility preservation is an emerging therapeutic approach that holds promise for child and adolescent cancer victims, though none of the proposed ovoprotective pharmaceutical agents have yet been implemented in clinical practice [12,65–73]. Ovoprotective drugs that have shown promise in animal studies include FTY720 (protects against radiation ovotoxicity), AS101 (protects against cyclophosphamide), imatinib and mesna (protect against cisplatin), bortezomib and Dexra (protect against DXR), and tamoxifen (protects against cyclophosphamide *in vivo*, DXR *in vitro*), and goserelin (protects against multi-drug breast cancer chemotherapy) [23,27,66,73–78]. The differences in mechanisms of action and specificity of chemotherapy protection may allow combined ovoprotective drug therapy to shield the ovary from multiple chemotherapeutic agents. Dexra and bortezomib represent the first proposed ovoprotective agents that specifically target mechanisms underlying chemotherapy-induced ovarian toxicity, and hold considerable promise for prevention of chemotherapy insult in girls and women. Bortezomib is a proteasome inhibitor and by binding the proteasome active site with high affinity and specificity, prevents proteasome-mediated active transport of DXR from the cytoplasm to the nucleus [79]. A combination of both bortezomib and Dexra would be expected to prevent DXR entry into the nucleus and inhibit the development of TOPII- mediated dsDNA breaks. Both Dexra and bortezomib have been studied at the single drug level to better define mechanisms mitigating DXR-induced ovarian toxicity [23,80]. Future studies will assess ovoprotection in the context of a multidrug approach using clinical chemotherapy cocktail protocols. Furthermore, the potential impact of ovoprotection therapy on long-term ovarian function remains to be determined in human subjects. Developing targeted ovarian delivery mechanisms may further reduce any side effects of ovoprotective agents and enhance their clinical application.

## Conclusions

Dexra mitigated acute DXR-induced ovarian toxicity and improved the fertility window as shown by increased fecundity, pup weight, litters size, and number of deliveries post-DXR therapy. The 1:1 Dexra:DXR dose conferred ovarian protection. Easy-to-administer Dexra may provide a timely, cost effective and safe, drug-based method for ovarian protection, particularly for prepubertal and adolescent girls for whom oocyte and embryo freezing are not viable fertility preservation options.

## Supporting Information

S1 Table Treatment arms in the study. The experimental design for the breeding study comprised eight independent treatment groups (total 157 mice). Additionally, 72 animals were used for the acute studies. The control, DXR, and bortezomib treatments were previously reported in [27].
(DOCX)

S1 Fig TUNEL-positivity induced by DXR is prevented by Dexra pretreatment. Whole ovarian tissue sections for Fig 3; zoomed images in Fig 3 are shown in full for control, DXR, and Dexra+DXR treatments as indicated. TUNEL signal in green, propidium iodide (nuclei) in red. Scale bar = 100 μm. The control and DXR-only treatment groups adapted from [27].
(TIF)
