# Supplementary material for: Dexrazoxane Diminishes Doxorubicin-Induced Acute Ovarian Damage and Preserves Ovarian Function and Fecundity in Mice
Source: PLoS One. 2015 Nov 6;10(11):e0142588. doi: 10.1371/journal.pone.0142588 (PMC4636352; doi:10.1371/journal.pone.0142588)
Supplement: S1 Table — The experimental design for the breeding study comprised eight independent treatment groups (total 157 mice). Additionally, 72 animals were used for the acute studies. The control, DXR, and bortezomib treatments were previously reported in [27]. (DOCX) [file pone.0142588.s001.docx]

|  | **Treatment** | **Total number of animals** |
| --- | --- | --- |
| **1.** | Vehicle for doxorubicin (DXR) and dexrazoxane (Dexra) | 16 |
| **2.** | DXR (10 mg/kg) + vehicle for Dexra | 16 |
| **3.** | Dexra (1 mg/kg) + vehicle for DXR | 12 |
| **4.** | Dexra (10 mg/kg) + vehicle for DXR | 12 |
| **5.** | Dexra (10 mg/kg) + DXR (10 mg/kg) | 21 |
| **6.** | Dexra (100 mg/kg) + DXR (10 mg/kg) | 16 |
| **7.** | Bortezomib (0.143 mg/kg) + vehicle for DXR | 32 |
| **8.** | Bortezomib (0.143 mg/kg) + DXR (10 mg/kg) | 32 |
