# Supplementary figures and images for: Dexrazoxane Diminishes Doxorubicin-Induced Acute Ovarian Damage and Preserves Ovarian Function and Fecundity in Mice
Source: PLoS One. 2015 Nov 6;10(11):e0142588. doi: 10.1371/journal.pone.0142588 (PMC4636352; doi:10.1371/journal.pone.0142588)

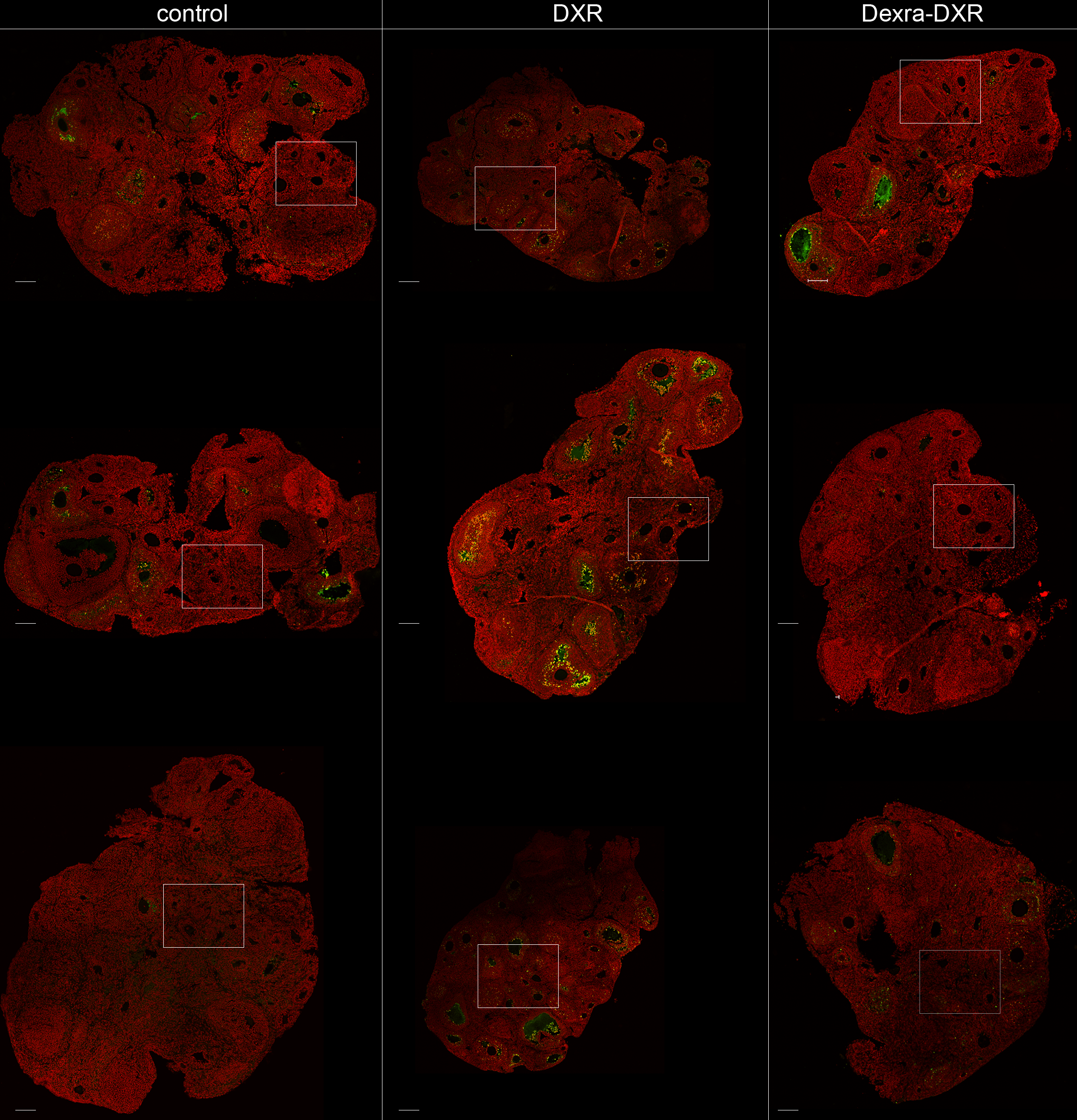

Supplement: S1 Fig — Whole ovarian tissue sections for Fig 3; zoomed images in Fig 3 are shown in full for control, DXR, and Dexra+DXR treatments as indicated. TUNEL signal in green, propidium iodide (nuclei) in red. Scale bar = 100 μm. The control and DXR-only treatment groups adapted from [27]. (TIF) [file pone.0142588.s002.tif]
